# Gender differences in the relationships between meaning in life, mental health status and digital media use during Covid-19

**DOI:** 10.1186/s12889-023-16672-x

**Published:** 2023-09-11

**Authors:** Wendy Wing Yan So, Bowie Po Yi Woo, Clifford Wong, Paul Siu Fai Yip

**Affiliations:** 1 The Hong Kong Jockey Club Centre for Suicide Research and Prevention, The University of Hong Kong, Pokfulam, Hong Kong; 2 Department of Social Work & Social Administration, The University of Hong Kong, Pokfulam, Hong Kong

**Keywords:** Digital media use, Meaning in life, Gender differences, Mental health, COVID-19

## Abstract

**Background:**

The COVID-19 pandemic has had a significant impact on individuals’ social lives, mental health status, and meaning in life (MIL). Globally, the use of different types of digital media has become a proxy for pre-COVID social lives for many people. This study investigated gender differences in the relationship between use of digital media, mental health status and MIL, during COVID-19 in Hong Kong.

**Methods:**

This cross-sectional study surveyed 1,488 young people recruited via city-wide random sampling in 2021. Respondents completed a phone survey on digital media use, Patient Health Questionnaire (PHQ-2), Generalized Anxiety Disorder (GAD-2), COVID-19 impact, meaning in life, and demographics. Gender differences in MIL were tested with an independent sample t-test. Gender-specific multiple linear regression models tested associations between MIL and explanatory variables of age, educational level, history of diagnosis, digital media use, and mental health status.

**Results:**

There was a significant gender difference in MIL (males (M = 12.90, SD = 4.12); females (M = 13.45, SD = 3.96); *t* (1485) = -2.656, *p* = .008). For males, all variables significantly associated to MIL (*F* (9, 759) = 15.731, *p* < .000, *R*^*2*^ = .157). However, for females, while the overall model for MIL was significant (*F* (9, 709) = 12.105, *p* < .001, *R*^*2*^ = .133), the only significant associated variable was mental health status.

**Conclusion:**

Females had significantly better MIL under COVID-19 than males. Digital media use contributed to MIL in males but not females, and there were gender-specific associated factors of MIL.

## Introduction

During the period of August 2, 2021, to December 17, 2021, Hong Kong faced sporadic outbreaks of COVID-19 cases, which included localized clusters and imported cases. To curb the spread of the virus and mitigate its impact on public health and the economy, the government implemented a range of measures. These measures encompassed the promotion of vaccination, strict border control measures, mass testing and contact tracing. In particular, many new policies, including reduction of dine-in services, closure of schools, implementation of work-from-home arrangements, etc. have been implemented to restrict social activities and limit spread of infection. Most people now stay at home, with a much-restricted social life, and consequently, many have become disconnected or isolated from others [1, 2].

Gender differences in reactions to COVID-19 have been observed, with studies showing differences in psychological responses between males and females [3, 4]. Laufer and Shechory Bitton found that females in particular experienced higher levels of anxiety, depression, and somatization due to isolation during COVID-19 [3]. Higher levels of resilience was found among men than among women [3]. Mental health status is an important predictor of meaning in life (MIL) [5–7]. MIL refers to the manner in which individuals feel their lives have purpose [8]. Steger et al. reported significant gender differences in MIL, with females having higher levels of the presence of, and searching for, MIL than males [9]. Pre-COVID-19, depression and anxiety were reported as negatively-correlated with MIL [8], and these findings hold true in the context of COVID-19 [10].

Moreover, recent findings suggested a negative relationship between interpersonal alienation and MIL during COVID-19 [11]. Interpersonal relationships have been widely regarded as a source of MIL [12–14]. According to the Meaning Maintenance Model (MMM), people have an inherent need to perceive events within a framework of mental representations that provide structure and meaning to their understanding of the world [15]. This drive is fulfilled by cognitive processes that apply pre-existing mental constructs to incoming information. When people encounter situations that challenge their existing mental constructs, such as a large discrepancy between actual and expected interpersonal relationships, they may experience an elevated level of interpersonal alienation that leads to a sense of meaninglessness. In other words, when our expectations of how our relationships should be are not met, it can disrupt our sense of meaning and purpose in life [16]. In other words, social isolation experienced during the pandemic may produce adverse effects on people’s development of MIL. One possible implication is that improving interpersonal connections may also improve people’s level of MIL. One feasible way during pandemic restrictions to alleviate social isolation is to increase the usage of virtual communication via digital media [17, 18].

The convenience and speed of information dissemination on social media has led to overwhelming news about COVID-19, such as reports on its prevalence, mortality rates, confirm cases, and high contagion, causing stress and heightened depression and anxiety [19, 20]. According to the study *Digital 2021 Hong Kong*, average total daily time spent on the internet by people in Hong Kong increased from 6 h 16 min in 2019 to 7 h 15 min in 2020 [21]. This report also indicated that Hongkongers aged from 16 to 64 years spend an average of two hours a day on social media in 2021 [21]. It is suggested that individuals have increasingly relied on digital media for social connection during the COVID-19 pandemic [22]. Nguyen et al. found that females were more likely to increase the amount of virtual communication by video calls and text messages, while males were more likely to do so over online games [23]. Another study indicates that among females, the frequent use of social media as a coping strategy is linked to higher levels of perceived negative impacts on academic performance and stress levels compared to males [24]. However, both genders experience similar negative mental health consequences with frequent social media use [24].

The growing body of research in this area suggest that mental health status is an important predictor of MIL [8, 25–29]. However, little attention has been paid to the effect of digital media use on MIL during COVID-19, when it was often the only viable way to communicate with friends and family because of social contact restrictions. Islam et al. suggest that problematic smartphone use (PSPU) and problematic social media use (PSMU) were significantly associated with adverse psychological well-being outcomes such as anxiety and depression amidst the ongoing pandemic, given that psychological well-being is a construct closely related to MIL as demonstrated by prior research [7, 30–32]. We identified only one study in the United States on social media use and MIL during the pandemic, which found no significant relationship [33]. No study has directly examined whether MIL is associated with digital media such as video communication apps (i.e., Zoom, Skype, Facetime, Microsoft Teams), or whether males and females use these apps differently.

This study aims to investigate gender differences in the relationship between use of digital media, mental health status and MIL, during COVID-19 in Hong Kong. Examining these gender differences in app utilization and the subsequent influence on MIL is essential for understanding the distinct coping strategies employed by each gender in response to social isolation. This knowledge can guide the development of more inclusive, gender-sensitive digital platforms tailored to the specific needs and preferences of both males and females. By addressing these factors, targeted interventions can be designed to bolster mental health and MIL, ultimately fostering improved mental health outcomes for both genders during the pandemic.

## Method

### Study design

This study is a cross-sectional study and was approved by the Human Research Ethics Committee for Non-Clinical Faculties of HKU (Research Ethics Approval ID: EA1709039).

### Participants

Permanent residents of Hong Kong aged between 18 to 35 years of age, were eligible to participate.

### Sample generation and procedures

Our data was a subset of the Hong Kong Mobile Phone Survey on Youth Mental Health and Internet Usage, initiated by the Hong Kong Jockey Club Centre for Suicide Research and Prevention (CSRP). From 2 August 2021 to 17 December 2021, a random sample of mobile phone numbers was generated using mobile number prefixes published by the Office of the Communications Authority (OFCA). For our study, a random sample of 125,746 mobile phone numbers was extracted. Potential respondents were contacted using the Computer-Assisted Telephone Interviewing (CATI) System, calling from 6:30 pm to 10:30 pm on weekdays. If the respondent was in the included age bracket, the study aim was described to potential participants as research to gain insights into the younger generation’s general well-being and usage of the internet. Participants gave informed verbal consent before completing the survey, and they understood that they could withdraw anytime. Should they experience distress during the survey, they were encouraged to seek help from emotional support services and hotlines, whose contact information was provided at the end of the survey.

### Measures

#### Demographics

Participants answered questions about age, gender and educational level, and a binary-response mental health question: *“Have you been diagnosed with major depressive disorder, schizophrenia, social phobia, or avoidant personality disorder?”*

#### Digital media use

This was measured as use of social media platforms (i.e., Facebook, Instagram, Twitter, Weibo, WeChat Moment) and video communication Apps (i.e., Zoom, Skype, Facetime, Microsoft Teams). The two binary-response questions were *“Consider your recent communication habits, have you ever used social media platforms to update personal status?”* and *“Consider your recent communication habits, have you ever used video communication apps to make video phone calls?”*

#### Patient Health Questionnaire 2-item (PHQ-2)

This is an ultra-brief self-report instrument for depression that contains the first two items of the PHQ-9 [34]. Each item in PHQ-2 asked about the frequency of a depressive symptom experienced in the last two weeks. The score for each item ranges from 0 (*never*) to 3 (*almost every day*), with a total score of 6. A cut off score of 3 is suggested as indicating a possible diagnosis of depressive disorder. Items were *“Little interest or pleasure in doing things*” and *“Feeling down, depressed or hopeless”.* In our current sample, Cronbach’s alpha of PHQ-2 was acceptable (0.61).

#### Generalized Anxiety Disorder 2-item (GAD-2)

It is an ultra-short screening tool that includes the first two items of GAD-7. Its scores are the same as the PHQ-2. Items were *“Feeling nervous, anxious or on edge”* and *“Not being able to stop or control worrying”*. A cut-off of 3 or greater is recommended in the general population to screen for GAD [34]. In our current sample, Cronbach’s alpha of GAD-2 was acceptable (0.75).

#### Impact of COVID-19

Emotional distress under COVID-19 was assessed by one question *“Have you been emotionally distressed by the COVID-19 pandemic?”* using a five-point scale ranging from 1 (*not at all*) to 5 (*very serious*).

#### Meaning in Life Questionnaire – Short Form (MLQ-SF)

Three items from the MLQ presence subscale were included in the study [35], *“My life has a clear meaning or purpose”*, *“I have found a satisfactory meaning in life”* and *“I have a clear sense of what gives meaning to my life.”* Items were rated from 1 (*Absolutely untrue*) to 7 (*Absolutely true*). The MLQ-SF was used in national health surveillance research in the United States, revealing good reliability and validity [36]. In our current sample, the Cronbach’s alpha of MLQ-SF was good (.85).

### Statistical analysis

All analyses were conducted utilizing IBM SPSS v. 25. Our analysis encompasses multiple stages to explore the relationships between our variables of interest.

First, we aimed to examine the difference in the level of Meaning in Life (MIL) between genders. To achieve this, we performed an independent sample t-test, a parametric test used to compare the means of MIL scores between two independent groups: males and females.

Next, a multivariate approach was used to investigate the relationships between our explanatory variables and MIL, separately for each gender. This was done through the construction of gender-specific multiple linear regression models. In our models, MIL served as the dependent variable, while age, educational level, history of diagnosis, digital media use (encompassing social media platform usage, chat application usage, and video communication app usage), and mental health status (as measured by PHQ-2 for depression, GAD-2 for anxiety, and a self-report measure for emotional distress under COVID-19) were the explanatory variables.

For the categorical variables of digital media use (social media platform and video communication apps), we utilized a binary coding scheme: “never use” was coded as 1 and “used” was coded as 2. This coding scheme allowed us to examine the effects of any usage versus non-usage of these digital platforms on MIL.

Significant factors in the models were identified using a 5% significance level. The effect size, direction, and statistical significance of each explanatory variable were reported. The overall fit of the model was also evaluated and reported using R^2^, which represents the proportion of the variance for a dependent variable that’s explained by the independent variables in the regression model. A larger value of R^2^ represents a larger proportion of the variance in the dependent variable can be predicted from the independent variables.

## Results

A total of 1,501 participants completed a community-based mobile survey on digital media use and general well-being. Data with missing values or from those who could not complete the questionnaire were excluded. The final sample consisted of 1,488 respondents. Of these, 51.7% (*n* = 769) were male and 48.3% (*n* = 719) were female. The mean age for males was 27.2 years (SD = 3.91), and for females, it was 27.2 years (SD = 3.85). The majority of both males (90.6%) and females (92.0%) had an educational level of post-secondary or above. Over 70% of both male and female participants had used social media platforms, while over 50% had used video communication apps. The prevalence of depressive symptoms and anxiety symptoms was 15.7% (Male = 58.5%) and 16.0% (Female = 51.7%) respectively during the COVID-19 outbreak.

Table 1 presents the results of a chi-square analysis comparing education level, history of diagnosis, social media platform use, and video communication app use across genders. Significant differences emerged between genders in the use of social media platforms (*p* < .001) and video communication apps (*p* < .001). However, no significant differences were observed in education level or history of diagnosis between males and females.

**Table 1:** Chi-square analysis comparing education level, history of diagnosis, social media platform use, and video communication app use by gender

	Male (*n* = 769)		Female (*n* = 719)		
	n	%	n	%	*P*
Education level					
Post-secondary or above	697	90.6	661	92.0	.490
Secondary School	69	9.0	57	7.9	
Primary School	2	0.3	0	0.0	
Refuse to answer	1	0.1	1	0.1	
History of Diagnosis					
Yes	46	6.0	58	8.1	.117
No	723	94.0	661	91.9	
Social Media Platform					
Used	585	76.1	628	87.3	< .001
Never Use	184	23.9	91	12.7	
Video Communication Apps					
Used	451	58.6	488	67.9	< .001
Never Use	318	41.4	231	32.1	

Table 2 delineates the findings of an independent-samples t-test comparing age, Meaning in Life (MIL), PHQ-2, GAD-2, and emotional distress under COVID-19 by gender. A significant gender difference in MIL levels was identified (*p* = .008). Young males exhibited significantly lower MIL scores (M = 12.90, SD = 4.12) compared to young females (M = 13.45, SD = 3.96). Additionally, a significant gender difference emerged in GAD-2 scores (*p* = .007). No significant differences were detected between males and females in terms of age, PHQ-2 scores, or emotional distress under COVID-19.

**Table 2:** Independent Sample t-test comparing age, meaning in life, PHQ-2, GAD-2, and emotional distress under COVID-19 by gender

	Male (*n* = 769)		Female (*n* = 719)		*P*	Cohen’s d
	M	SD	M	SD		
Age	27.2	3.91	27.2	3.85	.781	3.88
Meaning in Life	12.9	4.12	13.5	3.96	.008	4.04
PHQ-2	1.35	1.45	1.27	1.27	.232	1.36
GAD-2	1.31	1.44	1.51	1.30	.007	1.37
Emotional Distress under COVID-19	2.79	1.26	2.87	1.15	.185	1.21

For young males, the model explained a significant amount (15.7%) of the variance in MIL (*p* < .000, *R*^*2*^ = .157). All variables contributed significantly to the model (Table 3). For young females, the model also explained a significant amount of variance (13.3%) in MIL (*p* < .001, *R*^*2*^ = .133) (Table 4), however only PHQ-2 (*p* < .000), GAD-2 (*p* = .013) and emotional distress under COVID-19 (*p* = .037) contributed significantly to the model.

**Table 3:** Regression coefficients for factors associated with meaning in life under COVID-19 (Male samples)

Associated factors	*B*	*SE*	*β*	95% CI		*P*
				Lower	Upper	
Social Media Platform	.902	.332	.094	.250	1.554	.007
Video Communication App	.692	.285	.083	.133	1.251	.015
PHQ-2	-.546	.118	-.192	-.778	-.315	.000
GAD-2	-.305	.120	-.107	-.541	-.069	.011
Emotional Distress (COVID-19)	-.363	.111	-.111	-.580	-.146	.001
History of Diagnosis	-1.229	.604	-.071	-2.415	-.043	.042
Age	.109	.035	.104	.040	.178	.002
Educational Level	1.483	.434	.115	.630	2.336	.001

*N* = 769. Constant = 9.073, *F* (8, 760) = 17.642, *p* < .000, *R*^*2*^ = .157

*CI* Confidence interval for *B*

**Table 4:** Regression coefficients for factors associated with meaning in life under COVID-19 (Female samples)

Associated factors	*B*	*SE*	*β*	95% CI		*P*
				Lower	Upper	
Social Media Platform	.314	.424	.026	-.519	1.147	.459
Video Communication App	.472	.300	.056	-.117	1.060	.116
PHQ-2	-.769	.131	-.246	-1.027	-.511	.000
GAD-2	-.321	.128	-.106	-.573	-.070	.012
Emotional Distress (COVID-19)	-.265	.123	-.077	-.507	-.023	.032
History of Diagnosis	-.568	.524	-.039	-1.596	.460	.278
Age	.049	.037	.048	-.023	.121	.183
Educational Level	-.209	.479	-.015	-1.149	.731	.662

*N* = 719. Constant = 13.802, *F* (8, 710) = 13.551, *p* < .000, *R*^*2*^ = .132

*CI* Confidence interval for *B*

## Discussion

This paper reports the first known information on how digital media use contributed to MIL in young Hong Kong residents during Covid-19. Gender differences in MIL suggested that gender-specific models for understanding these associations were required. In males, usage of social media platforms, usage of video communication Apps, older age, and higher educational level positively affected MIL, whilst higher levels of depression and anxiety, higher levels of emotional distress under COVID-19, and previously-diagnosed mental health disorder negatively affected MIL. Two of the associated factors (use of social media platforms and video communication Apps), indicated that males who used them had higher MIL than males who did not. For females, the model explained slightly less of the MIL variance (13.3%) than for males, although similar to males, higher levels of depression and anxiety, and higher levels of emotional distress under COVID-19 negatively affected MIL. However, no variable positively associated with MIL for females, and no form of digital media use associated with MIL under COVID-19.

While mental health outcomes were significantly associated with of MIL in both genders, only males seemed to be benefited from digital media use, given that higher level of MIL correlated with lower state anxiety and lower COVID-19 stress [10]. One of the plausible explanations may be that the overuse of social media could produce negative effects that outweigh the beneficial effects of digital media use. A recent study found that females were more prone to overusing digital media during COVID-19, compared to males [37]. A recent study found that cyberbullying, lack of sleep, and lower physical activity mediated the link between digital media use and mental health among females but not among males [38]. Also, females may be more likely to engage in relational aggression and males in physical aggression, a difference seemingly exacerbated by media exposure [39–41]. These gender differences suggest that digital media use might have a more negative impact on females’ well-being than males. Furthermore, this study found significant gender differences in MIL. Digital media use was positively associated with MIL only for males, although MIL was associated with mental health for both genders. Future studies should attempt to measure and model more factors to increase understanding of the factors associated with MIL, particularly under pandemic conditions. Factors of interest could include life satisfaction, sense of purpose, intrinsic and extrinsic aspirations [25, 42].

This study presents a limitation in temporal specificity. The phrase ‘recent communication habits’ was used, but the precise time frame—intended to refer to the COVID-19 pandemic period—was not explicitly defined. This could have led to varied interpretations among respondents, potentially influencing the reliability of the responses. Future research should provide clear time frames to ensure more consistent understanding and improved data accuracy. Moreover, the cross-sectional study design precludes investigation of causal relationships between media use and MIL. Future studies should aim to test this using randomized controlled trials to compare the effects on MIL of different types of digital media. This study only targets young adults. During the pandemic, increased use of digital media became more common, even for elderly [43], thus further research is warranted to examine the use of digital media in different age groups, and its effect on MIL. It is imperative to acknowledge that numerous studies have established a correlation between excessive social media usage and negative effects on mental health [44–49]. However, our study adopted a binary approach (with or without) and did not consider the degree of social media use, which precludes a precise exploration of the impact of social media on mental health. In conclusion, based on our current findings, it remains inconclusive whether social media plays a positive role in fostering MIL during the pandemic. Further research is necessary to understand the extent to which social media affects mental health and to ascertain the appropriate measures that individuals can take to avoid any adverse consequences.

## Data Availability

The datasets used and/or analysed during the current study available from the corresponding author on reasonable request.

## Abbreviations

CSRP: Centre for Suicide Research and Prevention
CATI: Computer-Assisted Telephone Interviewing
GAD-2: Generalized Anxiety Disorder 2-item
MIL: Meaning in Life
MIQ-SF: Meaning in Life Questionnaire – Short Form
OFCA: Office of the Communications Authority
PHQ-2: Patient Health Questionnaire 2-item
SPSS: Statistical Package for the Social Sciences
